# Valorization of Fruit and Vegetable Waste: An Approach to Focusing on Extraction of Natural Pigments

**DOI:** 10.3390/foods14081402

**Published:** 2025-04-18

**Authors:** Khadija Ramzan, Syeda Hijab Zehra, Aiste Balciunaitiene, Pranas Viskelis, Jonas Viskelis

**Affiliations:** Lithuanian Research Centre for Agriculture and Forestry, Institute of Horticulture, Kaunas Str. 30, Kaunas District, 54333 Babtai, Lithuania; hijab.zehra@lammc.lt (S.H.Z.); aiste.balciunaitiene@lammc.lt (A.B.); pranas.viskelis@lammc.lt (P.V.)

**Keywords:** natural pigments, valorization of food waste, extraction techniques, biodegradable polymers

## Abstract

The increasing demand for functional foods has spurred interest in bioactive compounds, particularly their role in health promotion and disease prevention. This review comprehensively explores the bioavailability, mechanisms of action, and potential applications of bioactive compounds derived from natural food sources. We have systematically compiled and synthesized data from the recent scientific literature, including peer-reviewed journal articles, clinical studies, and meta-analyses, to present an in-depth evaluation of these compounds’ physicochemical properties, stability, and interactions within food matrices. Furthermore, this review discusses advanced delivery systems, such as nanoencapsulation and emulsification, for enhancing bioavailability and targeted release. By addressing critical gaps in the understanding of the functional and technological aspects of bioactive compounds, this review underscores their relevance in formulating novel nutraceuticals and functional foods. The insights presented herein provide a foundation for future research and practical applications in the food industry, ultimately contributing to improving human health and well-being. Although recovering bioactive compounds from food waste is a sustainable way to reduce waste and use resources, additional research is required to make these procedures more efficient for use on an industrial scale.

## 1. Introduction

Agro-industries usually generate fruit waste (byproducts), which are generally exploited as residual biomasses rich in a variety of bioactive functional components. Fruit wastes are inherently rich in phytochemicals, and several facets, such as their fiber, phenolic compounds, and other bioactive components, have been extensively studied. Fruit byproducts primarily consist of peels, seeds, and pomace, which serve as valuable reservoirs of bioactive compounds with notable nutritional and functional properties. These include proteins, carbohydrates, polysaccharides, and a diverse range of secondary metabolites [1]. Plants contain natural pigments, which generate the bright colors of fruits and vegetables, along with other types of food made from plants, during their production. The four main plant pigments, namely, anthocyanins, betalains, carotenoids, and chlorophylls, are secondary metabolites. They also make food visually attractive and provide health benefits, including antioxidant functions and anticancer abilities. The interest in natural pigments continues to rise because consumers recognize the risks synthetic colorants pose in food, including as allergens and their potential toxicity, despite their use due to their low cost, high stability, and strong color properties [2].

The modern shift among consumers toward purchasing products that have clean labels and are environmentally friendly, alongside possessing health-promoting features, has increased the market demand for natural alternatives to synthetic food additives. Researchers and food industry personnel have examined sustainable natural pigments as a result of this development [3]. The market has started to explore fruit and vegetable waste products consisting of peels and seeds, along with pomace and pulp residues, as valuable but underused resources. Large quantities of byproducts are generated during processing, yet many industry operators discard them, thus generating pollution and economic waste [4].

The valuation of agricultural waste contributes to waste reduction and circular economic practices and leads to economical methods of obtaining bioactive natural compounds from food waste [5]. The extraction of food waste pigments supports global sustainability initiatives because it enhances the functional properties of processed foods, leading to better nutritional value and sensory benefits. The commercial use of pigments extracted from agro-industrial byproducts is subject to technological and scalability barriers that constrain these pigments’ full-scale application [6]. This evaluation of fruit and vegetable waste valorization methods explores in detail the extraction of natural pigments while paying special attention to this specific process throughout the research [7]. This paper explains the different food waste pigments as well as their therapeutic impacts and functional uses alongside recent technological advances toward better pigment extraction methods. This publication places emphasis on this sustainable method while also demonstrating that natural pigments serve a dual purpose, acting both as food additives and beneficial ingredients that shape the future of human health along with food science development [8,9].

The extraction of high-value bioactive compounds, including antioxidants, phenolic compounds, essential oils, polysaccharides, lignin, and proteins, from food waste and byproducts enhances resource utilization and supports the development of more sustainable biorefinery approaches [10,11]. This review critically examines the major classes of natural pigments present in food waste, explores advanced extraction methodologies, and discusses encapsulation strategies aimed at improving pigment stability. Additionally, it highlights the broad-spectrum applications of these pigments across various industries, emphasizing recent advancements in environmentally sustainable processes for obtaining bioactive natural colorants from food waste.

## 2. Pigments in Agricultural Byproducts

**Figure 1:**
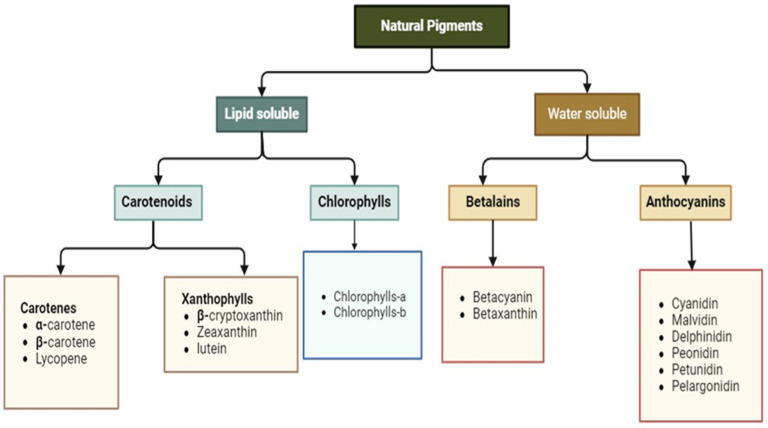
Classification of pigments on the basis of their solubility.

Pigments are categorized as either natural or synthetic and distinguished by their solubility in fat and/or water. They can also be classified as inorganic or organic. They are also divided into groups based on their natural occurrence, solubility, and structural affinities. The classification of natural pigments, primarily extracted from plant-derived waste materials, is illustrated in Figure 1. These pigments are primarily categorized into four principal groups: betalains, anthocyanins, carotenoids, and chlorophylls.

Pigments are classified based on various criteria, including their origin (synthetic or natural), solubility (fat-soluble, water-soluble, or both), and chemical composition (organic or inorganic). Additionally, they can be divided into groups based on their natural occurrence, solubility traits, and structural attributes. Figure 1 is a modified diagram of [12]. Four main categories can be used to classify natural pigments made from plant waste: betalains, anthocyanins, carotenoids, and chlorophyll.

### 2.1. Anthocyanins

**Figure 2:**
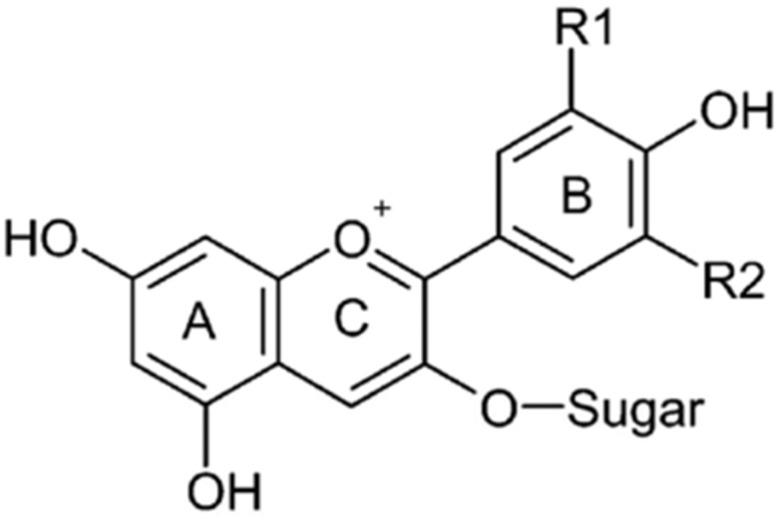
General structure of an anthocyanin.

Anthocyanins constitute a major class of water-soluble, non-toxic, vacuolar pigments within the polyphenol family (Figure 2). Over 700 distinct anthocyanin structures are known to exist [12]. Fruits and vegetables that are high in anthocyanins include red radishes, red cabbage, purple sweet potatoes, black carrots, cherries, blackcurrants, and a variety of berries, including concord grapes, blackberries, elderberries, black raspberries, chokeberries, black goji berries, and blueberries [13].

The class of phenolic compounds includes the water-soluble secondary metabolites known as anthocyanins. They exist as glycosides of anthocyanidins, that is, the aglycone form. The primary anthocyanidins present in food include delphinidin, cyanidin, pelargonidin, malvidin, petunidin, and peonidin [14]. These compounds are abundant in various fruits and vegetables, like blueberries, blackberries, figs, cranberries, grumixama, grapes, saffron petals, jucara, and purple corn. Byproducts and waste generated from food-processing industries, particularly the wine and juice sectors, represent rich sources of anthocyanin pigments, which may be used as natural food coloring in a variety of ways [15]. For example, the high anthocyanin concentration (4.31 mg of Cy3GlE/g) in blackberry residues makes them important sources of natural colorants and nutraceuticals. Aronia, a native North American berry, also exhibits a substantial concentration of anthocyanins [16].

To date, anthocyanins have been found to be the most potent, secure, and organic water-soluble free radical scavengers. Their potential application in the pharmaceutical sector as alternatives to synthetic pigments, such as lemon yellow, indigo, and carmine, is being explored. These synthetic pigments are frequently used as drug dyes but may pose health risks due to their regular consumption [17].

### 2.2. Betalains

**Figure 3:**
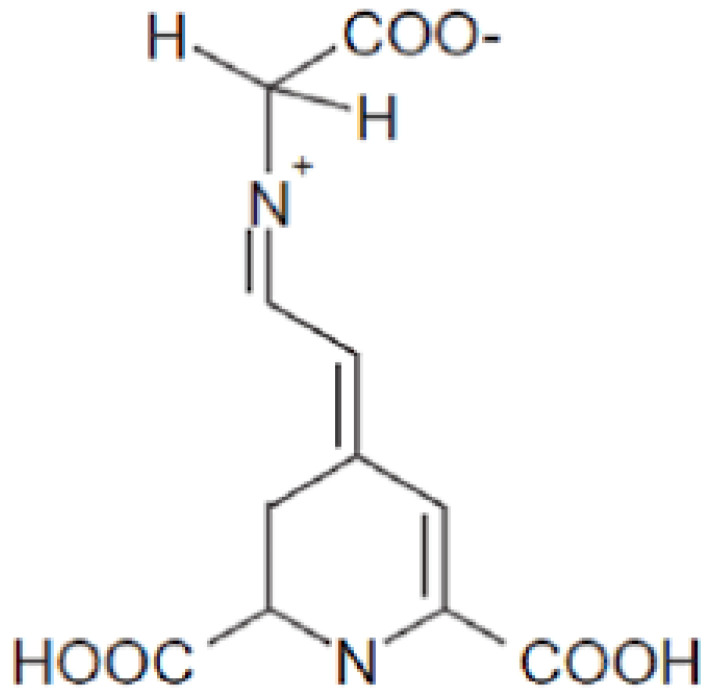
General skeleton structure of a betalain.

Betalains are a class of water-soluble nitrogenous pigments that impart color to a limited range of plants. These pigments are derived from betalamic acid [4-(2-oxoethylidene)-1,2,3,4-tetrahydropyridine-2,6-dicarboxylic acid]. When betalamic acid condenses with amines, it can produce betaxanthins, which are yellow pigments. Alternatively, when it condenses with imino compounds, such as cyclo-DOPA and its derivatives, it can produce betacyanins, which are violet pigments (Figure 3) [18,19]. Significant sources of betalains include plants like amaranth, pitaya fruit, prickly pears, and beetroot. The varying color phenotypes of these plants correlate with their respective concentrations of betaxanthins and betacyanins. Depending on species, cultivar, and growing conditions, beetroot can have concentrations of 3754–11,932 mg/kg dry weight, making it one of the most plentiful natural sources of betalains [20,21]. More than 70% of the world’s beetroot is produced in Europe, making it the leading producer worldwide. The United Kingdom, in particular, generates significant amounts of beetroot waste due to its substantial beetroot juice industry [22]. The primary factors influencing the stability of betalains throughout storage and food preparation are pH and temperature. Betanin, for instance, has demonstrated stability for a minimum of 20 days at 4 °C and more than 275 days at −30 °C when stored at pH 7. A significant challenge limiting the physiological efficacy of betalains is their limited bioavailability. Additionally, betalains exhibit a relatively narrow color spectrum [21].

### 2.3. Carotenoids

**Figure 4:**
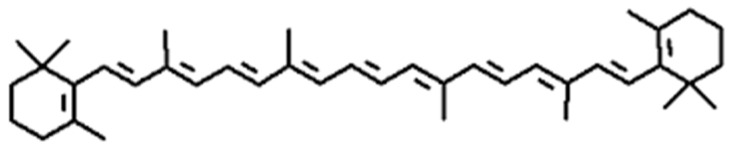
General skeleton structure of beta-carotene.

Carotenoids, a class of tetraterpenoid pigments, give many fruits and vegetables their vivid yellow, orange, and red colors. These C40 compounds are categorized into xanthophylls and carotenes (Figure 4). Carotenes are polygenic hydrocarbons, whilst xanthophylls are oxygenated derivatives of carotenes, often formed through hydroxylation or epoxidation reactions [11]. In nature, carotenoids are abundant, especially in vegetables and fruit, and produced by plants and algae. Over 1000 different carotenoids exist. Due to their lipid solubility, carotenoids are readily absorbed and accumulated in the fatty tissues of animals, as seen in salmon, flamingos, lobsters, and other organisms [20].

Carotenoids are sensitive to heat, and thermal processing can significantly reduce their extraction yields. The extracted carotenoids, as natural pigments, can improve the color properties of food products, such as French fries, and potentially reduce reliance on synthetic colorants. Carotenoids are a valuable source of natural colorants, serving as a safer and more sustainable alternative to synthetic pigments [15]. Unlike synthetic pigments, which may release harmful chemicals, carotenoids are generally recognized as safe (GRAS). However, their lipid solubility can pose challenges in incorporating them into hydrophilic food matrices. Emulsification techniques can be employed to overcome this limitation and enhance the bioavailability of carotenoids in various food products [23].

### 2.4. Chlorophyll

**Figure 5:**
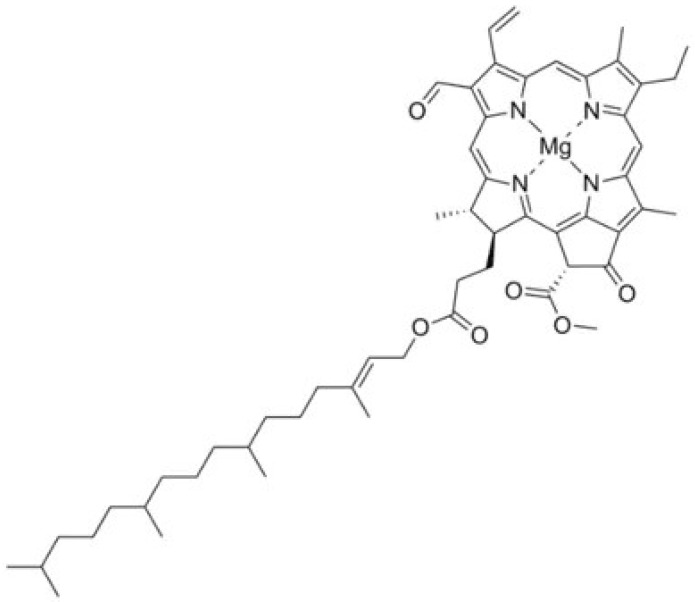
The skeleton structure of chlorophyll a.

Chlorophylls are amphiphilic pigments, characterized by a hydrophilic porphyrin head and a hydrophobic phytol tail. Because of their lipophilic nature, chlorophylls are typically insoluble in polar solvents [24]. The two main types of chlorophyll found in plants are chlorophyll-a and chlorophyll-b. The two forms differ structurally by a single functional group at the 7-carbon position: chlorophyll-a has a methyl group (-CH3), while chlorophyll-b has an aldehyde group (-CHO) [25,26]. This structural variation results in distinct color hues, with chlorophyll-A appearing blue-green (Figure 5) and chlorophyll-B appearing yellow-green [27].

A common food coloring, chlorophyll is a naturally occurring green pigment that satisfies EU criteria as E140. Its long history of consumption in plant-based foods has established its safety as a food additive. However, its volatility limits its application as a colorant [28]. While chlorophyll can be extracted from various plant sources, including plant waste, there is a scarcity of research exploring the use of plant waste for chlorophyll extraction and its subsequent application as a food colorant. As consumer awareness of natural colorants and their health benefits grows, the demand for chlorophyll is increasing [29].

## 3. Extraction Techniques

The use of synthetic solvents to extract natural pigments has been the subject of numerous investigations. However, this Section focuses on environmentally friendly extraction methods. Table 1 outlines several methods of green extraction, including microwave-assisted extraction (MAE), ultrasound-assisted extraction (UAE), supercritical fluid extraction (SFE), pressurized liquid extraction (PLE), pulsed-electric-field-assisted extraction (PEF), and enzyme-assisted extraction (EAE), which have been used to recover natural pigments from plant waste and remnants [30,31,32,33].

Green extraction methods frequently make use of environmentally benign solvents such glycerol, ethanol, water, ionic liquids (ILs), fatty acid esters, vegetable oils (such as soybean, rapeseed, and cocoa oil), and NADES. These solvents are gaining increasing attention for their potential use in natural color extraction. Many researchers are exploring the use of green solvents and alternative organic techniques to extract natural pigments [32].

**Table 1:** Overview of green extraction methods used for isolating pigments from plant-derived waste materials.

Source	Scientific Name	Extraction Techniques	Processing Conditions	Yield (per 100 g/d.w) and Pigment Extracted	References
Apple peel	*Malus domestica*	Solvent extraction method	80% acetone or ethanol	169.8 g cyanidin 3-glucoside	[34]
Egg plant peel	*Solanum melongena*	Solvent extraction method	70% methanol, acetone, and ethanol	82.84 mg of methanol, 62.93 mg of ethanol, 51.55 mg of acetone	[35]
Red grape pomace	*Vitis vinifera*	Water extraction	Hot water	41–68% monomeric anthocyanins	[36]
Egg plant peel	*Solanum melongena*	Water extraction	Water (80 C, 40 min)	-	[37]
Blackberry residue	*Rubus* sp.	Solvent extraction method	Acidified ethanol and citric acid	4.32 mg of cyanidin 3-glucoside	[38]
Coffee exocarp	*Coffea aps.: C. Arabica and C. robusta*	Ethanol extraction	60% ethanol	0.145 mg of cyanidin 3-glucoside	[39]
Banana peel	*Musa* sp.	Solvent extraction method	Ethanol, methanol, water, and acetone	434 μg of cyanidin 3-glucoside	[40,41]
Grape extracts	*Vitis vinifera*	Supercritical CO_2_ extraction	30–40 °C,100–130 bar, Ethyl alcohol, 25–50 mL/min CO_2_ flow rate	80–85% recovery of cyanidin 3-glucoside	[42]
Grape products	*Vitis vinifera*	Combined extraction	Water temperature: 70 °C, ultrasound: 35 KHz, PEF (3 KV cm^−1^)	7.93, 7.76, and 4.05 mg of methanol, acetone, and ethanol, respectively	[43]
Grape skin	*Vitis vinifera*	Solvent extraction method	Acidified 0.01% HCl 70% acetone along with chloroform (evaporated at 30 °C)	987.8–382.1 mg of Mv3G	[44]
Grape extracts	*Vitis vinifera*	Supercritical CO_2_ extraction	45–46 °C,160–165 kg/cm^2^, 6% ethanol	1.176 mg/ml	[45]
jaboticaba peel	*Plinia cauliflora*	Pressurized liquid extraction + supercritical CO_2_	5 MPa, 80 °C, 9 min in CO_2_, in the presence of ethanol	-	[46]
Black currant waste	*Ribes nigrum*	Water extraction	Acidified water extraction via solid-phase extraction (SPE)	2% *w*/*w* of dry weight	[47]
jaboticaba peel	*Plinia caulifora*	Ultrasound-assisted extraction	Hydroalcoholic mixture, ultrasonic bath (40 KHz and 150 W)	3.4 mg/g raw material	[48]
Black currant byproducts	*Ribes nigrum*	Water extraction	Water	718.47–389.0 mg of cyanidin 3-glucoside	[49]
Jaboticaba pomace	*Plinia caulifora*	Ultrasound-assisted extraction	water, 531 W CM-2, 20 °C, 15 min	510.35 mg of cyanidin 3-glucoside	[50]
Red grape	*Vitis vinifera*	Combined extraction	water and ethanol UAE-water/ethanol (1:1), 25 KHz, 300 W, 20 °C, 60 min MAE-water/ethanol (1:1), 200 W, 50 °C, 60 min	UAE-34,188 ppm	[51]
Red pitaya peel	*Hylocereus polyrhizus*	Solvent extraction	80% acetone	13.8 g betanin	[52]
Prickly pear pericarp	*Opuntia joconostle*	Solvent extraction	80% methanol + 0.1% HCl	4.55 mg betanin	[53]
Ulluco peel	*Ullucus tuberosus*	Solvent extraction	Methanol/water (60:40), 10 °C, 24 h	100 μg/g	[54,55]
Beetroot	*Beta vulgaris*	Ultrasound-assisted extraction	50% aqueous ethanol/0.5% acetic acid. 50–60 Hz, 125 W, 22 C	peel-3.8–7.6 mg/g pomace-37.22 mg/100 g	[56]
Dragon fruit	*Hylocereus undatus*	Ultrasound assisted extraction	80% acetone, 15 min	101.04 mg/100 g	[57]

**Figure 6:**
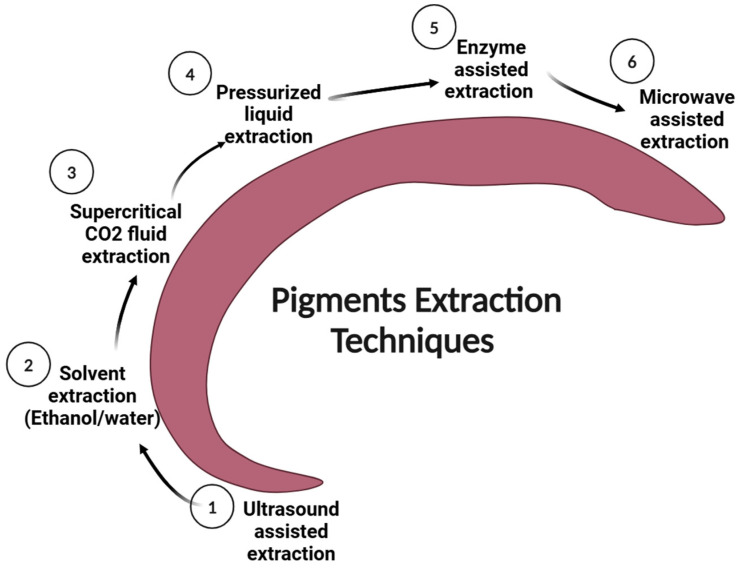
Various extraction techniques are suggested or frequently utilized for the extraction of natural pigments from plant waste.

Figure 6 presents various advanced extraction techniques utilized for the recovery of natural pigments [12]. These approaches are sought after due to their numerous advantages, including their minimal solvent requirements, high speed, convenience, increased extraction yields, ability to protect pigments from degradation, ability to enhance natural colorant quality, and greater eco-friendliness compared to conventional extraction methods [58] (Table 2).

### 3.1. Ultrasound-Assisted Extraction

Ultrasound-assisted extraction (UAE) is a non-thermal method that relies upon the formation of cavitation bubbles induced by ultrasonic vibrations. Cavitation enhances mass transfer by disrupting cell walls and releasing intracellular components into the solvent [26,59]. This technique works especially well for removing substances that are susceptible to heat. However, temperature control may be necessary for extended UAE treatments, performed by using cooling systems, such as water baths or condensers [60,61,62]. Several strategies have been explored to scale up UAE processes and achieve continuous extraction. For instance, patents exist for large-scale ultrasonic reactors with capacities of 1 m^3^ [63]. Additionally, ultrasound probes can be integrated into flow-through systems or placed at the ends of crushing or grinding devices to increase the effectiveness of extraction [64].

### 3.2. Microwave-Assisted Extraction

Microwave-assisted extraction (MAE) enables quick and even heating by combining microwave energy with traditional solvent extraction. Microwaves, unlike conventional heating techniques, heat materials selectively, providing accurate and effective temperature control [65]. Solvent penetration, temperature, and solute solubility are all influenced by microwave power, which increases pigment yield and shortens extraction times. However, high temperatures have the potential to harm heat-sensitive pigments; thus, it is important to carefully optimize process variables, including sample matrix and treatment time [66,67,68]. Additionally, the polarity of the solvent employed plays a crucial role in microwave absorption and subsequent heating. Polar solvents absorb microwave radiation more efficiently, accelerating the extraction process [69]. As a result, MAE is particularly effective for extracting polar compounds but may have limitations for heat-sensitive pigments [66].

### 3.3. Enzyme-Assisted Extraction

Enzyme-assisted extraction (EAE) encourages hydrolysis via enzymes, which breaks down cell wall polysaccharides. Cell wall disintegration can be accelerated by the combined action of many enzymes, improving extraction efficiency [70]. This non-thermal method enables highly specific reactions under mild conditions, preserving the stability of extracted pigments [71,72]. Cellulases, pectinases, and hemicellulases are the primary enzymes used for extracting secondary metabolites [59]. Despite the advantages of EAE, its widespread application is hindered by several limitations, including the low stability, in the long run, of the enzymes used under the operational conditions (extreme pH and elevated temperature); challenges in recovering the mixture of the reaction; and the difficulty of reusing enzymes [3].

### 3.4. Pressurized Liquid Extraction (PLE)

Pressurized liquid extraction (PLE) entails using high pressure to keep a solvent liquid at temperatures higher than its typical boiling point. This technique reduces the surface tension and viscosity of a solvent [70,73]. The elevated temperature facilitates the solvent’s diffusion into the matrix and speeds up the food waste structure’s disintegration. Based on which solvent is selected, PLE can be employed to extract both nonpolar and polar pigments [70]. The primary advantages of PLE include its rapid extraction time, low solvent consumption, inert extraction conditions (under nitrogen atmosphere), and lack of requirement for additional filtration [74]. However, temperature-sensitive chemicals cannot be extracted with PLE, and it can result in high operating expenses [59].

### 3.5. Supercritical Fluid Extraction

Supercritical fluid extraction (SFE) is a solvent extraction method in which chemicals are extracted from solid matrices using supercritical fluids like carbon dioxide and nitrogen. A supercritical fluid exists at a temperature and pressure above its critical point, featuring characteristics both of a liquid and a gas. This unique state allows for enhanced solvent power and improved solubility of organic compounds, facilitating the process of extracting bioactive substances like pigments [75,76].

High efficiency, quick extraction periods, and lower solvent usage are just a few of the benefits of SFE. The use of non-polar solvents like carbon dioxide minimizes the risk of solvent contamination in the extracts. Additionally, SFE is considered a safe and environmentally friendly method since it allows the use of renewable solvents [77].

However, since most supercritical fluids are non-polar, SFE works especially well with lipid-soluble pigments like chlorophylls and carotenoids. The high pressure requirements of SFE can lead to significant capital and operational costs [26].

**Table 2:** Advantages and limitations of modern techniques for extracting plant pigments.

Extraction Method	Advantages	Disadvantages	References
Ultrasound-assisted extraction	It is characterized by its versatility, flexibility, low cost, and ease of use. It facilitates rapid energy transfers and minimizes solvent usage. It requires short extraction times (ranging from 5 to 60 min). It can be coupled with thermal treatments to enhance yields or enzymatic treatments to increase anthocyanin yields and the bioactivity of extracts. It is scalable for large-scale applications.	Process homogeneity is enhanced by the probe system (PUE). Large-scale applications may be constrained by increased costs and process nonlinearity. Post-extraction, filtration and clean-up steps are necessary. The procedure may result in operator fatigue.	[78,79,80]
Microwave-assisted extraction	It provides rapid and uniform heating It involves minimal solvent consumption. There are short extraction times (1–40 min). Recent advancements in vacuum microwave extraction have resulted in a microwave-assisted extraction (MAE) method with reduced reactor temperatures. It has potential for large-scale application.	The solvent must be microwave-absorbent. Heating may compromise the structure and activity of certain compounds. Post-extraction, filtration and clean-up steps are necessary.	[65]
Pulsed-electric-field extraction	The extraction time is very brief. This process can be conducted at room temperature. It involves low energy and monetary costs and is suitable for large-scale application.	Some compounds may be susceptible to high electric fields. It is advantageous to decrease the electrical conductivity of the matrix before extraction. In industrial applications, challenges include the non-uniform distribution of electric pulses, the limited availability of suitable solvents, and the need for a cooling system to regulate temperature when extracting thermolabile compounds under high-electrical-pulse conditions.	[81,82]
Supercritical fluid extraction	In this method, CO_2_, which is easily removable post-extraction and minimizes thermal degradation, is used as a solvent. The extraction time is up to 1 h; it does not require an additional energy source It is scalable for employment in large-scale applications.	It requires a co-solvent to extract polar compounds. The quantity and type of co-solvent must be optimized along with other parameters. Subcritical water extraction (SWE) necessitates high temperatures to achieve subcritical conditions, though ethanol can be used as an alternative to water.	[83,84,85]
High-hydrostatic-pressure extraction	It involves a short extraction time (~5 min) and can be performed at room temperature for higher repeatability. It requires smaller amounts of solvents. It is suitable for possible large-scale applications.	There are high initial investment costs and maintenance/service costs. High pressure could impact the structure or activity of certain compounds. Parameters need to be optimized to mitigate these effects.	[86]
Pressurized liquid extraction	It features low solvent consumption and protects oxygen- and light-sensitive compounds. It requires specific temperature conditions. It is suitable for large-scale application.	It requires costly equipment and necessitates a clean-up step post-extraction. The extraction duration ranges from 1 to 2 h.	[87]
High-voltage electrical discharge	This process can be carried out at low temperatures, with short extraction times and minimal energy input It is suitable for large-scale applications.	It involves high maintenance and service costs. High-voltage electrical discharges may generate chemical products and free reactive radicals, potentially affecting the bioactive activity of antioxidants. Providing an MAE (microwave-assisted extraction) method with lower reactor temperatures could mitigate these issues, enabling possible large-scale applications.	[81]
Enzyme-assisted extraction	It involves the use of moderate extraction conditions that are eco-friendly and selective due to enzyme specificity. It can be combined with ultrasonic extraction to enhance the yield and bioactivity of the extract.	The high cost of enzymes, variability in enzyme activity based on pH, temperature, and nutrient conditions of the matrix pose limitations. Post-extraction, it requires filtration and clean-up steps. There are difficulties in scaling due to long extraction times (1–12 h), limited availability of commercial enzyme types, and occasional low selectivity and variability.	[88,89,90]

## 4. Enzymatic Degradation of Pigments: Mechanisms and Implications

Pigments such as chlorophylls, betalains, carotenoids, and anthocyanins play fundamental roles in the visual appeal, nutritional value, and physiological functions of plant-based foods. These pigments contribute to color intensity, antioxidant activity, and health-promoting properties. However, during post-harvest storage, processing, and food formulation, enzymatic degradation can significantly impact pigment stability, leading to undesirable changes in color, bioactivity, and shelf-life. The enzymatic breakdown of these pigments is mediated by specific enzyme systems that catalyze oxidative, hydrolytic, and other biochemical reactions. Understanding these degradation pathways is crucial for designing strategies to enhance pigment stability in food products and optimize storage conditions [91].

### 4.1. Enzymatic Degradation of Chlorophylls

**Figure 7:**
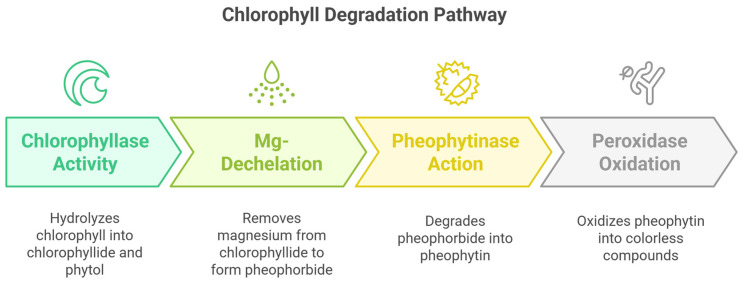
Enzymatic degradation of chlorophyll.

Chlorophylls are highly susceptible to enzymatic degradation, leading to their breakdown and the formation of pheophytins, pyrochlorophylls, and colorless catabolites (Figure 7). Chlorophyllase (EC 3.1.1.14) is the primary enzyme responsible for hydrolyzing chlorophyll, converting it into chlorophyllide and phytol. This process is further influenced by peroxidases, which catalyze the oxidation of chlorophyll derivatives, leading to pigment loss [91]. The enzymatic degradation of chlorophylls is often accelerated by post-harvest conditions such as temperature fluctuations, pH changes, and exposure to oxygen, constituting a major challenge in preserving the green color of vegetables and leafy greens [92].

### 4.2. Enzymatic Breakdown of Betalains

**Figure 8:**
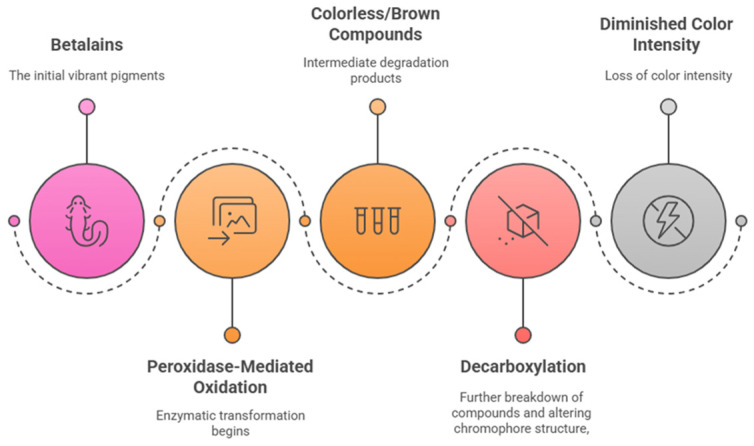
Betalin degradation sequence.

The stability of betalains is highly dependent on enzymatic degradation pathways, primarily involving peroxidases and polyphenol oxidases (PPOs). Peroxidases catalyze the oxidative cleavage of betalains, leading to color fading and reduced bioactivity. Additionally, glycosidase enzymes can hydrolyze sugar moieties attached to betalain molecules, further destabilizing their structures [93,94]. Betalains’ enzymatic degradation is explained in Figure 8.

### 4.3. Enzymatic Oxidation of Carotenoids

The enzymatic degradation of carotenoids primarily involves lipoxygenases (EC 1.13.11.12), which catalyze oxidative cleavage, leading to the formation of volatile compounds and color loss. Carotenoid cleavage dioxygenases (CCDs) are another class of enzymes that target the central double bonds in carotenoid molecules, producing apocarotenoids and further diminishing their color intensity. This enzymatic breakdown is particularly significant in food-processing environments where oxidative stress conditions are prevalent. Strategies for mitigating carotenoid degradation include the use of antioxidants, modified-atmosphere packaging, and low-temperature storage [94,95].

### 4.4. Degradation of Anthocyanin by Enzymes

**Figure 9:**
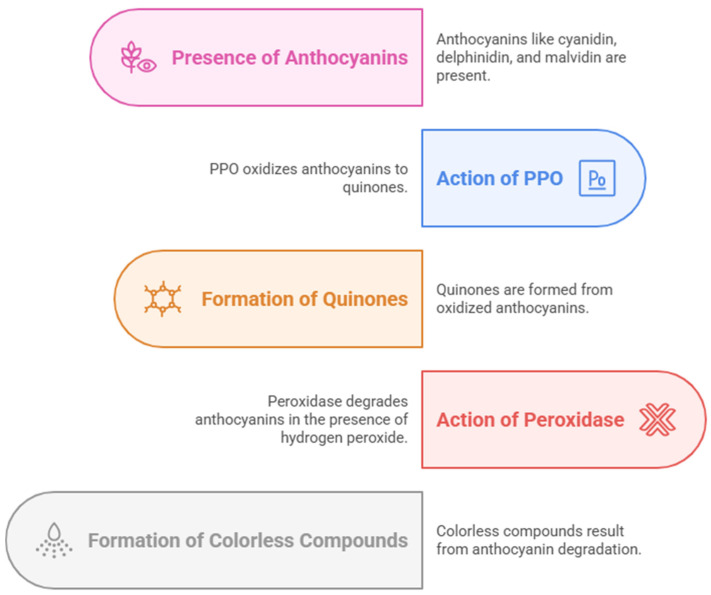
The enzymatic degradation of anthocyanins.

Anthocyanins are highly susceptible to enzymatic degradation, particularly by polyphenol oxidases (PPOs) and peroxidases (Figure 9). PPOs catalyze the oxidation of anthocyanins, leading to the formation of quinones and subsequent polymerization reactions that result in color loss and browning. Additionally, β-glucosidase enzymes hydrolyze anthocyanins by cleaving sugar moieties, making the anthocyanin aglycones more prone to oxidation and degradation. Environmental factors such as pH, light exposure, and metal ion interactions further influence the stability of anthocyanins. To enhance anthocyanin retention in food matrices, approaches such as co-pigmentation, microencapsulation, and enzyme inhibition strategies have been explored [96,97].

## 5. Non-Enzymatic Degradation of Pigments: Mechanisms and Implications

Pigments’ stability is significantly influenced by environmental factors, leading to non-enzymatic degradation that affects the color retention, bioactivity, and overall quality of plant-based foods. Understanding the mechanisms underlying the degradation of these pigments is critical for food preservation, post-harvest technology, and the development of stable food products. The non-enzymatic degradation of pigments is a complex phenomenon influenced by multiple environmental factors, leading to significant color and bioactivity loss in plant-based foods. Understanding the mechanisms underlying pigment degradation provides a foundation for developing innovative strategies for enhancing pigment stability [94].

### 5.1. Chlorophyll Degradation

Chlorophylls are green pigments essential for photosynthesis. Their degradation occurs through multiple pathways, predominantly influenced by light, oxygen, pH, and temperature. The primary mechanism of non-enzymatic chlorophyll degradation involves the oxidation and dephytylation of chlorophyll molecules. Under acidic conditions, chlorophylls convert into pheophytins due to the replacement of the central magnesium ion with hydrogen, leading to color loss. Additionally, exposure to high temperatures accelerates chlorophyll degradation by promoting oxidative cleavage, resulting in the formation of colorless catabolites. Light exposure induces photo-oxidation, wherein singlet oxygen and free radicals initiate structural modifications that lead to pigment breakdown [98].

### 5.2. Betalain Degradation

Betalains are nitrogen-containing water-soluble pigments classified into red-violet betacyanins and yellow betaxanthins. Unlike anthocyanins, betalains are highly sensitive to environmental factors such as temperature, pH, oxygen, and water activity. The primary non-enzymatic degradation mechanism of betalains involves oxidation, hydrolysis, and thermal degradation. Oxidative degradation is facilitated by the presence of metal ions and free radicals, leading to pigment bleaching and a loss of color intensity. High temperatures accelerate betalain degradation through decarboxylation and dehydrogenation reactions, rendering food products less visually appealing. Moreover, exposure to alkaline conditions causes structural modifications, reducing pigment stability [94,99].

### 5.3. Carotenoid Degradation

Carotenoids, including β-carotene, lycopene, and lutein, are lipid-soluble pigments with significant antioxidant properties. Their stability is highly influenced by light, oxygen, and heat, leading to non-enzymatic degradation via oxidation, isomerization, and polymerization. Oxidative degradation is the primary pathway, initiated by singlet oxygen and free radicals, resulting in the breakdown of the conjugated double-bond system, thereby reducing color intensity and bioactivity. Isomerization occurs under thermal stress, where trans-carotenoids are converted into cis isomers, which exhibit lower bioavailability and diminished antioxidant potential. In food processing, carotenoid degradation is exacerbated by prolonged heating and exposure to UV radiation, necessitating strategies such as encapsulation and antioxidant fortification to enhance pigment stability [100].

### 5.4. Anthocyanin Degradation

Anthocyanins are water-soluble flavonoid pigments responsible for red, blue, and purple hues in various plant tissues. Their stability is strongly dependent on pH, temperature, light exposure, and the presence of copigments. The non-enzymatic degradation of anthocyanins occurs primarily through oxidation, thermal degradation, and structural rearrangements [101,102]. At higher temperatures, anthocyanins undergo cleavage of their glycosidic bonds, leading to the formation of unstable aglycones that further degrade into colorless or brown products. Oxidative degradation is facilitated by the presence of metal ions and ascorbic acid, which act as pro-oxidants, accelerating pigment loss. Additionally, pH-induced structural transformations cause anthocyanins to shift between different ionic forms, where alkaline conditions promote color fading. Light exposure triggers photodegradation, leading to the formation of chalcones and subsequent polymerization reactions that diminish pigment stability. Strategies such as copigmentation, encapsulation, and pH optimization are commonly employed to mitigate anthocyanin degradation in food products [96,97].

## 6. Stabilization of Natural Pigments

Stability is an important factor when evaluating the application of natural pigments as colorants and antioxidants in food. Natural pigments, including carotenoids, chlorophylls, and anthocyanins, frequently exhibit poor stability as a result of their susceptibility to a variety of environmental factors, including oxygen, light, pH, and temperature. As a result, these pigments are susceptible to deterioration during extraction and storage procedures [103]. To alleviate such deterioration, the food business utilizes diverse encapsulation procedures, which entail wrapping the bioactive component (core) with a protective material (encapsulant) [104]. This encapsulation can generate various capsule morphologies, such as spheres, films, or irregular particles, resulting in structures that are crystalline, porous, compact, or amorphous [105,106]. These encapsulated systems can release their active constituents under regulated conditions, thus enhancing the bioactive compounds’ stability and bioavailability. The selection of an appropriate encapsulation material is crucial to the efficacy of these encapsulation techniques; it must complement the active ingredient’s physical and chemical characteristics, the chosen encapsulation process, the necessary particle size, and the intended use [107,108,109].

Freeze-drying is extensively used in the food sector because of its ease of use, adaptability, and scalability. This method is particularly effective for encapsulating heat-sensitive substances, such as chlorophylls, carotenoids, and anthocyanins. Freeze-drying aids in maintaining the encapsulated material’s original characteristics, such as its biological activity, shape, size, flavor, appearance, texture, and color [110,111,112]. Another common encapsulation technique is thermal gelation, where the active ingredient is dispersed as small droplets in an aqueous medium and then surrounded by a gelled protective wall [113,114]. This method is cost-effective and straightforward, utilizing various encapsulating agents, such as pectins. Pectins are effective in soluble solutions at pH levels between 2.8 and 3.5. Another adaptable substance that is utilized in the food business as a thickening, gelling, stabilizing, and microencapsulating agent is calcium alginate [115,116].

Conversely, the emulsion technique involves combining two immiscible liquids into a relatively homogeneous mixture through the action of an emulsifier. Recently, this technique has been modified to encapsulate bioactive substances, allowing for regulated release while shielding the substances from deterioration and unwanted interactions, thereby enhancing their functionality and bioavailability [117]. Another advanced encapsulation method involves the formation of liposomes, which are vesicle structures composed of phospholipid bilayers. These lipid bilayers self-assemble into stable, spherical structures, shielding the hydrophobic lipid components from the aqueous environment. Liposomes can range in size from nanometers to micrometers. Liposome encapsulation offers numerous advantages, including enhanced stability and the potential for large-scale manufacturing with natural resources. The food industry makes extensive use of liposomes in both industrial and research settings. Methods for preparing liposomes include both mechanical and non-mechanical techniques. Mechanical techniques such as high-pressure homogenization, sonication, extrusion, colloidal milling, and micro-fluidization are commonly used. Non-mechanical techniques, including detergent removal and reverse-phase evaporation, are also employed [82,118].

## 7. Applications of Natural Pigments

Natural pigments are widely used within the food sector as colorants to restore and maintain the original color of food products, which can degrade during processing and storage. By integrating natural colors, the quality and consistency of fruits, which may be affected by environmental factors like weather and climate change, can be preserved. This not only enhances the aesthetic appeal of the food products but also contributes to improving their flavor and overall quality. Beyond the food industry, there are several applications for natural pigments in industries like medicines and nutraceuticals, where they serve as coloring agents, food preservatives, and quality indicators [8,13]. Below is a thorough explanation of these various applications.

### 7.1. Food Preservatives

Plant-based natural colorants are abundant in phytochemicals, which have antioxidant and antibacterial qualities that can inhibit microbial growth and reduce food spoilage [119]. Turmeric, a natural colorant known for its vibrant yellow hue, is a prime example. Due to its potent antibacterial and antioxidant activities, turmeric is widely used as a preservative in products like pickles and various food items. It is particularly rich in phenolic compounds and flavonoids, with curcumin being the primary phenolic component. Curcumin is highly effective at scavenging peroxyl radicals, contributing significantly to its preservative efficacy [120].

### 7.2. Color as a Quality Indicator

Food color is a key factor in assessing the quality of the final product and significantly influences customers’ perceptions of attributes such as flavor, sweetness, and freshness [103]. The anthocyanin profile, for example, can serve as a reliable indicator of authenticity, allowing consumers to distinguish between genuine and artificial berry products. Additionally, red cabbage extract’s bluish-red color, which is ascribed to betanin, is remarkably stable at high pH levels and can be used as a useful quality indicator [121].

### 7.3. Dietary Supplements

Colorants derived from botanical sources often contain bioactive components produced by plant cells, contributing to their potential health benefits [122]. One example is vitamin B12, or riboflavin, which is naturally found in various foods such as milk, leafy green vegetables, fish, and poultry [123]. Carotenoids represent yet another significant class of natural colors. These lipid-soluble pigments, which produce yellow, orange, and red hues, are widely distributed in plants and, to a lesser extent, certain animals [124].

### 7.4. Bioactive Properties and Health Advantages

**Figure 10:**
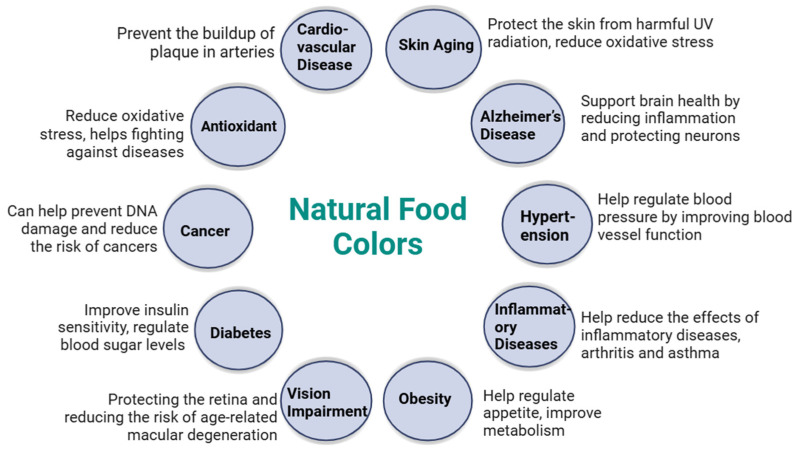
Effect of natural food colorants on human health.

In the food industry, pigments are utilized not only as color enhancers but also as additives and antioxidants. There is growing interest in exploring the use of various plant-based waste resources to extract food-grade pigments with bioactive properties. Growing consumer knowledge of the health advantages of natural compounds is provoking this interest (see Table 1, Figure 10). These pigments have many biological functions that support human health, such as cytokine-signaling-mediated anti-inflammatory actions [125], antioxidant properties through free radical scavenging [126], antimicrobial activity, anti-cancer activity, and antithrombotic and cardioprotective effects mediated by the mitogen-activated protein kinase pathway. Additionally, they promote neural and ocular health and help prevent non-communicable illnesses through mechanisms such as the cyclo-oxygenase pathway [127,128].

Natural colorants may offer significant health benefits due to their bioactive components, which possess medicinal properties such as strong antioxidant, antimutagenic, anti-arthritic, and anti-inflammatory activities [129]. Astaxanthin, a naturally occurring xanthophyll, exhibits potent antioxidant properties. Carotenoids, a class of lipid-soluble pigments, act as powerful biological antioxidants, protecting organs, tissues, and cells from oxidative damage caused by free radicals, and may have potential as anti-tumor agents [130]. Betacyanin is another antioxidant with strong anti-radical activity [131]. Furthermore, epidemiological research has connected consuming chlorophyll to a lower risk of colon cancer [132]. Numerous pigments from microbes with antioxidant qualities have also been discovered, such as carotenoids, anthocyanins, and violacein [133].

### 7.5. Extracts with Natural Pigments Used for the Green Synthesis of Metal Nanoparticles

The green synthesis of metal and metal oxide nanoparticles using plant extracts has gained attention as an eco-friendly alternative to traditional methods [134]. Plant extracts contain various biomolecules, such as alkaloids, flavonoids, and terpenoids, that act as reducing and stabilizing agents in the synthesis process [135]. This approach offers several advantages, including simplicity, cost-effectiveness, and a lack of hazardous chemicals. The synthesis process can be conducted at room temperature and easily scaled up [136]. Commonly synthesized nanoparticles include silver, gold, zinc oxide, and iron oxide. These green-synthesized nanoparticles have diverse applications, particularly in environmental remediation, such as microbe elimination, catalysis, pollutant removal, and heavy-metal sensing. The size and morphology of the nanoparticles can be influenced by various parameters during the synthesis process [137].

Anthocyanins are natural pigments found in plant-based sources. Recently, their significance in nanoparticle synthesis, including for silver, gold, and iron oxide nanoparticles, has attracted considerable attention due to their ability to act as reducing and stabilizing agents [138]. This reduction process involves the transfer of electrons from anthocyanins to metal ions, facilitating the formation of stable nanoparticles with controlled sizes and shapes [139]. Anthocyanins offer several advantages over conventional chemical agents, including biocompatibility, non-toxicity, and sustainability [140]. Anthocyanins exhibit strong reducing and stabilizing properties, enabling the synthesis of various MNPs, including silver, gold, and iron oxide nanoparticles [141]. The electron-rich structures of anthocyanins, particularly their hydroxyl and phenolic groups, play a crucial role in the bio-reduction of metal ions into nanoparticles [142]. During this process, anthocyanins donate electrons to the metal ions, reducing them to their nanoparticulate form while preventing aggregation through the stabilization of the nanoparticle surface. This green synthesis approach is advantageous as it avoids the use of toxic chemicals, reduces environmental impacts, and produces biocompatible nanoparticles suitable for applications in food, medicine, and environmental remediation [143].

### 7.6. Deposition of Pigments in Plastics

Pigments are insoluble colorants dispersed in plastics, preferred over dyes due to their superior fastness properties, especially with respect to migration resistance. The plastics industry has made progress in developing pigments with improved properties, including surface modifications to enhance compatibility with polymers [144]. Recent research has investigated the potential release of nanoscale pigments from plastics during environmental aging, food contact, and leaching. Results show a negligible release of pigments across various scenarios, with upper limits of 10 mg/m^2^ or 1600 particles/mL, indicating that pigment nanomaterials remain strongly contained in plastics like HDPE [145]. However, quantitative information about organic pigment migration in plastics has been sought to better understand the physical parameters governing such phenomena.

The integration of pigments into plastic matrices is essential for developing food packaging materials with enhanced visual appeal and functional properties. Pigments, whether organic or inorganic, are embedded within polymers to provide color, opacity, and UV protection [146]. Recent advancements focus on sustainable practices, including the use of pigments that improve recyclability, such as near-infrared (NIR)-detectable pigments that facilitate sorting during recycling processes [147]. Nanoparticle-based pigments, due to their high surface area, offer superior dispersion, color intensity, and stability; however, their environmental and toxicological impacts require comprehensive evaluation [148]. A significant challenge in food-packaging applications is the migration of pigment constituents into food, which is regulated by stringent safety standards to ensure consumer health. The stability and performance of pigments depend on their interaction with the polymer matrix, influenced by particle size, concentration, and surface modifications. Innovations in pigment technology now prioritize eco-friendly formulations, ensuring minimal environmental impact while maintaining functionality [149]. These developments support the broader transition toward circular economy principles in packaging. Research continues to explore biodegradable and bio-sourced pigments, optimizing their compatibility with polymers to meet the increasing demand for sustainable food-packaging solutions [150,151,152,153].

## 8. Conclusions

Globally, the increasing volume of food waste, driven by industrial food production to meet rising consumer demand, has become a pressing concern. Fruit and vegetable byproducts, which constitute a substantial fraction of this waste, represent a valuable resource for natural pigments, including chlorophylls, carotenoids, betalains, and anthocyanins. The incorporation of these natural pigments into food products not only enhances their visual appeal but also offers potential health benefits. Consequently, the extraction of natural pigments from food waste holds significant environmental and economic importance. However, conventional extraction methods are often labor-intensive, cost-intensive, and environmentally unsustainable. Additionally, natural pigments are highly susceptible to degradation when exposed to the elevated temperatures and prolonged processing times associated with traditional extraction techniques. To address these limitations, various innovative food-processing technologies have been developed to optimize pigment extraction. Techniques such as high-pressure-assisted extraction, ultrasound-assisted extraction, pulsed-electric-field extraction, and microwave-assisted extraction have demonstrated numerous advantages, including improved extraction efficiency, selectivity, reduced energy consumption, and a lower environmental impact, making them promising solutions for extracting sustainable pigments from food waste.

## Data Availability

No new data were created or analyzed in this study. Data sharing is not applicable to this article.
